# A MEMS Electrochemical Angular Accelerometer with Silicon-Based Four-Electrode Structure

**DOI:** 10.3390/mi15030351

**Published:** 2024-02-29

**Authors:** Mingbo Zhang, Qinghua Liu, Maoqi Zhu, Jian Chen, Deyong Chen, Junbo Wang, Yulan Lu

**Affiliations:** 1 State Key Laboratory of Transducer Technology, Aerospace Information Research Institute, Chinese Academy of Sciences, Beijing 100190, China; zhangmingbo21@mails.ucas.ac.cn (M.Z.); liuqinghua21@mails.ucas.ac.cn (Q.L.); zhumaoqi21@mails.ucas.ac.cn (M.Z.); chenjian@mail.ie.ac.cn (J.C.); dychen@mail.ie.ac.cn (D.C.); jbwang@mail.ie.ac.cn (J.W.); 2 School of Electronic, Electrical and Communication Engineering, University of Chinese Academy of Sciences, Beijing 100049, China

**Keywords:** microelectromechanical system, electrochemical angular accelerometer, rotational sensor, silicon-based four-electrode structure

## Abstract

This paper presents a MEMS electrochemical angular accelerometer with a silicon-based four-electrode structure, which was made of thousands of interconnected microchannels for electrolyte flow, anodes uniformly coated on structure surfaces and cathodes located on the sidewalls of flow holes. From the perspective of device fabrication, in this study, the previously reported multi-piece assembly was simplified into single-piece integrative manufacturing, effectively addressing the problems of complex assembly and manual alignment. From the perspective of the sensitive structure, in this study, the silicon-based four-electrode structure featuring with complete insulation layers between anodes and cathodes can enable fast electrochemical reactions with improved sensitivities. Numerical simulations were conducted to optimize the geometrical parameters of the silicon-based four-electrode structure, where increases in fluid resistance and cathode area were found to expand working bandwidths and improve device sensitivity, respectively. Then, the silicon-based four-electrode structure was fabricated by conventional MEMS processes, mainly composed of wafer-level bonding and wafer-level etching. As to device characterization, the MEMS electrochemical angular accelerometer with the silicon-based four-electrode structure exhibited a maximum sensitivity of 1458 V/(rad/s^2^) at 0.01 Hz and a minimum noise level of −164 dB at 1 Hz. Compared with previously reported electrochemical angular accelerometers, the angular accelerometer developed in this study offered higher sensitivities and lower noise levels, indicating strong potential for applications in the field of rotational seismology.

## 1. Introduction

Seismic motions consist of both translational and rotational components, resulting in vibrations with six degrees of freedom [1,2]. However, traditional seismic measurements only focus on translational components without paying attention to rotational aspects, which thus cannot fully meet the requirements for seismic resistance [3]. In fact, in recent years, a series of seismic damages caused by rotational vibrations have been recorded [4,5,6,7]. Due to the lack of effective measurements for angular accelerations, especially for angular accelerometers with low frequencies down to 0.01 Hz, the understanding of rotational components remains limited.

Angular accelerometers for seismic motion monitoring are sensors that convert low-frequency angular accelerations from external environments into electrical signals. The potential application is to monitor and prevent the hazards caused by the rotational components of seismic motions and to comprehensively analyze the causes of earthquakes. Current angular accelerometers for measuring seismic rotational components can be classified into three types based on solid inertial masses, sensitive microstructures and liquid inertial masses, respectively. Angular accelerometers based on solid inertial masses [8,9,10] had limited low-frequency performances and bulky sizes due to their wheel servo systems. Angular accelerometers based on sensitive microstructures [11,12,13] utilized sensitive principles of piezoelectric or piezoresistive effects enabled by microstructures (e.g., microcolumns or microbeams), and they were susceptible to linear vibrations and had limited frequency ranges and accuracies. Angular acceleration sensors based on liquid inertial masses [14,15,16] operated in a low-pass manner with high electromechanical conversion coefficients, demonstrating strong potential for detecting seismic motions with low amplitudes at low and ultra-low frequencies. The sensors working under the mechanism of electrochemical reaction have the merits of good adaptability [2] and controllability, and they are also highly sensitive to the ion concentration, thus achieving high sensitivity.

Traditional angular accelerations based on liquid inertial masses (electrochemical angular accelerometers) relied on platinum mesh electrodes as sensitive units [14], which suffered from the key problem of low consistency due to the misalignments among multiple layers of electrodes. Furthermore, the insulation layers of platinum meshes were made of porous ceramics, in which the production process was complicated and parameter adjustment accuracy was limited, further limiting measurement sensitivities. Therefore, optimizing the structure design and fabrication process of the electrode chip is key to the development of an electrochemical angular accelerometer. Since MEMS offered advantages such as miniaturization [2], integration, and mass production [17], a few pioneering studies were conducted to replace traditional platinum mesh electrodes with microfabricated electrodes, where both the sensitivities and working bandwidth of electrochemical angular accelerometers were significantly improved. However, these previously developed angular accelerometers with microfabricated micro-electrodes suffered from electrode structure and inter-electrode wires because of the planar setup of sensitive microelectrodes. The previously reported MEMS electrochemical angular accelerometers mostly comprise a silicon-based three-electrode structure [15] and a planar electrode structure [16]. Among them, the ones with a silicon-based three-electrode structure have higher sensitivity. But the chip did not have an active ion depletion layer, which would lead to unstable differential output. The parameters of an MEMS electrochemical angular accelerometer based on a planar electrode could be precisely adjusted. However, the small cathode area and large substrate area result in a low utilization efficiency of the on-chip area, limiting the sensitivity level within the frequency band.

This paper developed a MEMS electrochemical angular accelerometer to monitor the rotational components of seismic motions. A unique silicon-based four-electrode structure was included, which was made of thousands of interconnected microchannels for electrolyte flow, anodes uniformly coated on wafer surfaces, and cathodes located on the sidewalls of flow holes. From the perspective of device fabrication, the previously reported multi-piece assembly was simplified into single-piece integrative manufacture. The chip innovatively adopted a wafer-level bonding method based on SU-8, effectively addressing the problems of complex assembly and manual alignment. SU-8 serving as an intermediate layer existed around each hole and acted as both an insulation layer between the top and bottom cathodes and an ion depletion layer, which enabled stable output. From the perspective of the sensitive structure, in this study, the silicon-based four-electrode structure featured complete insulation layers between anodes and cathodes. The cathodes located on the sidewalls of flow holes were connected to each other by conduction through the silicon substrate, eliminating complex leads and improving the utilization efficiency of the on-chip area, which can enable fast electrochemical reactions with improved sensitivities.

## 2. Structure and Working Principle

**Figure 1:**
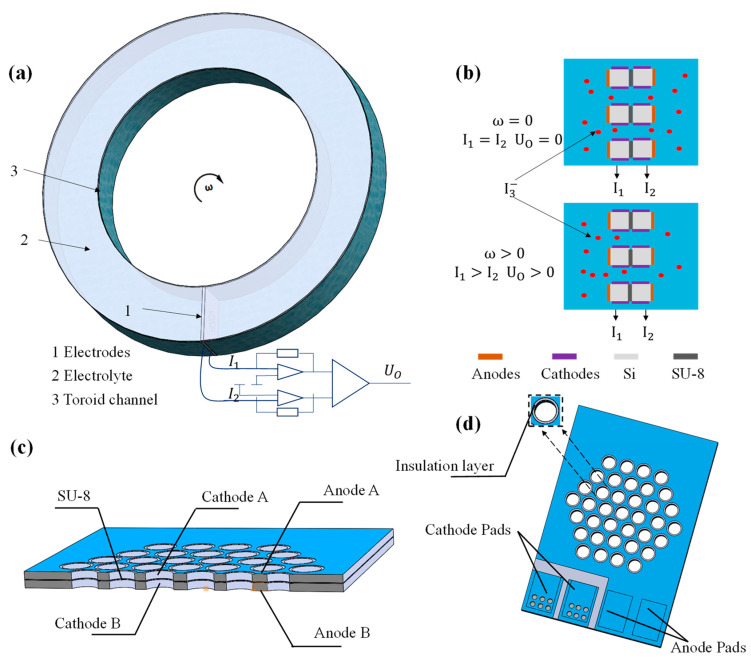
(**a**) The schematic of the MEMS electrochemical angular accelerometer with a silicon-based four-electrode structure, consisting of sensitive electrodes, a circular flow channel, and an electrolyte solution. (**b**) When the accelerometer detects external angular vibrations, there exists relative motions between the liquid inertial mass and the electrodes, where the $I_{3}^{-}$ concentration near the two cathodes changes in magnitude but with opposite directions, leading to current outputs. (**c**) The cross-section profile, and (**d**) the 3D schematic view of the silicon-based four-electrode structure which is based on two silicon wafers containing thousands of through-hole microchannels for the electrolyte solution. The sidewalls of the flow holes and the surfaces of the bonded silicon wafers are uniformly sputtered with Ti/Pt alloy as cathodes and anodes, respectively.

The MEMS electrochemical angular accelerometer based on the silicon-based four-electrode structure is illustrated in Figure 1a. It consists of sensitive electrodes, a toroid channel, and an electrolyte solution. The toroid channel is filled with the electrolyte solution composed of $I_{2}$ and KI. The sensitive electrodes are fixed inside the toroid channel, where two identical pairs of electrodes are arranged in an A-C-C-A (anode-cathode-cathode-anode) configuration, separated by insulation layers.

Due to the presence of the complexation reaction ${\;I}_{2}+I^{-}⇌I_{3}^{-}$, ${\;I}^{-}$ and $I_{3}^{-}$ in the electrolyte solution, these are the main components involved in the electrochemical reactions. During the operation of the electrochemical angular accelerometer, a high potential is applied to the surface-sensitive electrodes, which are regarded as two anodes, while a low potential is applied to the sensitive electrodes on the sidewalls of the flow holes, which function as two cathodes. When a voltage difference exists between anodes and cathodes, the following electrochemical reactions (anode: $3I^{-}-2e^{-}\to {\;I}_{3}^{-}$, cathode: $I_{3}^{-}+2e^{-}\to \;3I^{-}$) occur.

The working principle of the MEMS electrochemical angular accelerometer is illustrated in Figure 1b. When the accelerometer does not detect any external angular vibrations, there is no relative motion between the electrolyte and the sensitive electrodes, resulting in a stable distribution of $I_{3}^{-}$ near the sensitive electrodes. The output currents of the two cathodes are equal ($I_{1}=I_{2}$), and the differential output signal is zero (U_O_ = 0). When the MEMS electrochemical angular accelerometer detects external angular vibrations, it induces relative motions between the inertial liquid mass and the electrodes. Through convection and diffusion effects, the concentrations of $I_{3}^{-}$ near-two cathodes change in the same magnitudes but in opposite directions, causing the output current to change with direction ($I_{1}>I_{2}$).

The cross-section profile of the silicon-based four-electrode structure is shown in Figure 1c, which is based on two bonded silicon wafers containing thousands of through-hole microchannels for the electrolyte solution. The sidewalls of the flow holes and the surfaces of the bonded silicon wafers are uniformly sputtered with Ti/Pt alloy as cathodes and anodes, respectively. More specifically, cathodes are electrically connected through the silicon substrate with resistance less than 0.0015 Ω/cm, and external connectivity is maintained by sputtering metal after etching flow holes. The anodes are directly connected to silicon surfaces, and anode-cathode insulation is realized by the gap between anodes and cathodes.

The three-dimensional schematic of the silicon-based four-electrode structure is shown in Figure 1d. Each side has two wire-bonding pads for the same electrode, with a total of eight wire-bonding pads on the top and bottom sides, resulting in four electrodes. The wire bonding pads are connected to the signal acquisition circuit through wire bonding, and the silicon-based four-electrode structure is encapsulated in a casing for testing. The bonding between the silicon wafers is completed during the wafer fabrication process, where SU-8 serves as an intermediate layer and acts as both an insulation layer between the top and bottom cathodes and an ion depletion layer.

## 3. Theory and Numerical Simulation

The relative motions between the electrolyte solution and the silicon-based four-electrode structure follow the principle of fluid mechanics, and the electrochemical reactions near the sensitive electrodes adhere to Faraday’s law. Therefore, flow resistance and cathode area are key parameters that influence the performance of the MEMS electrochemical angular accelerometer [18,19]. Since the numbers and diameters of flow holes and cathode length determine the flow resistance and cathode area of this angular accelerometer, they are treated as crucial geometrical parameters in the design and optimization of the silicon-based four-electrode structure.

Since theoretical analysis can only provide a qualitative rather than quantitative relationship between the aforementioned geometrical parameters and the performance of the MEMS electrochemical angular accelerometer, numerical simulations were conducted in this study for structural optimization based on numerical results.

**Figure 2:**
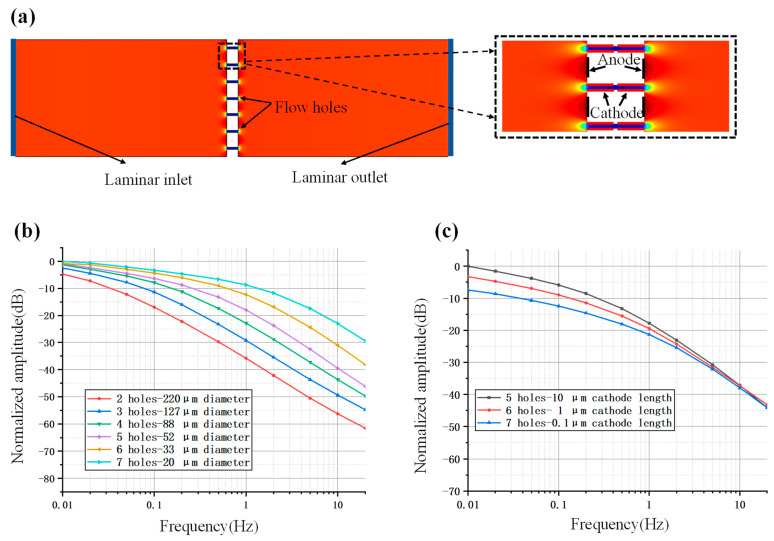
Numerical Simulation of the MEMS electrochemical angular accelerometer with silicon-based four-electrode structure. (**a**) A two-dimensional simulation is established based on the transverse cross section near the electrodes of the electrochemical angular accelerometer, where the simulation model includes the sensitive unit, its adjacent flow channels, and electrode boundary conditions. Simulation results for the silicon-based four-electrode structure with the influence of (**b**) different fluid resistance on the bandwidth and (**c**) different cathode area on the sensitivity where increases in fluid resistance and cathode area were found to expand working bandwidths and improve device sensitivity, respectively.

In COMSOL, the simulation model included the sensitive unit, its adjacent flow channels, and the internal electrolyte. The equations in fluid mechanics and Faraday’s law were applied, and the properties of incompressible fluid, ion transports and electrode boundary conditions were properly defined. Based on these settings, a two-dimensional simulation was established based on the transverse cross section near the electrode of the electrochemical angular accelerometer, as shown in Figure 2a.

Figure 2b shows simulation results for the influence of different fluid resistances on the bandwidth of the silicon-based four-electrode structure. The parameters used for simulation are shown in Table 1. More specifically, under a unit length with a cross section of 500 μm, a variety of number-diameter parameters of flow holes (e.g., 2 hole-220 μm of diameter, 3 hole-127 μm of diameter, 4 hole-88 μm of diameter, 5 hole-52 μm of diameter, 6 hole-33 μm of diameter, 7 hole-20 μm of diameter) were simulated for comparison under the unchanged cathode length of 100 μm for each hole. As to the simulation results, it was found that increases in flow resistance produced accelerometers with increased working bandwidth due to the turning point of the low-pass link shifting to the right, and the attenuation of the mid- to high-frequency curve slowing down.

Figure 2c shows the simulation results for the influence of different cathode areas on the sensitivity of the silicon-based four-electrode structure. More specifically, under a unit lengths with a cross section of 500 μm, under predefined number-diameter parameters of flow holes (e.g., 5 hole-52 μm of diameter, 6 hole-33 μm of diameter, 7 hole-20 μm of diameter), cathode length of 10 μm, 1 μm and 0.1 μm were simulated for comparison. As to the simulation results, it was found that increases in cathode length produced accelerometers with increased sensitivities due to increased rates of electrochemical reactions.

The simulation provides a basis for determining the effects of two key parameters, fluid resistance and cathode area, on device performance, which is crucial for chip parameter design and device selection. However, since two-dimensional simulations do not align perfectly with actual characteristics, devices with different parameters will be tested during actual fabrication. Parameters will be optimized and analyzed based on the conclusions drawn from the simulations.

## 4. Fabrication and Packaging

**Figure 3:**
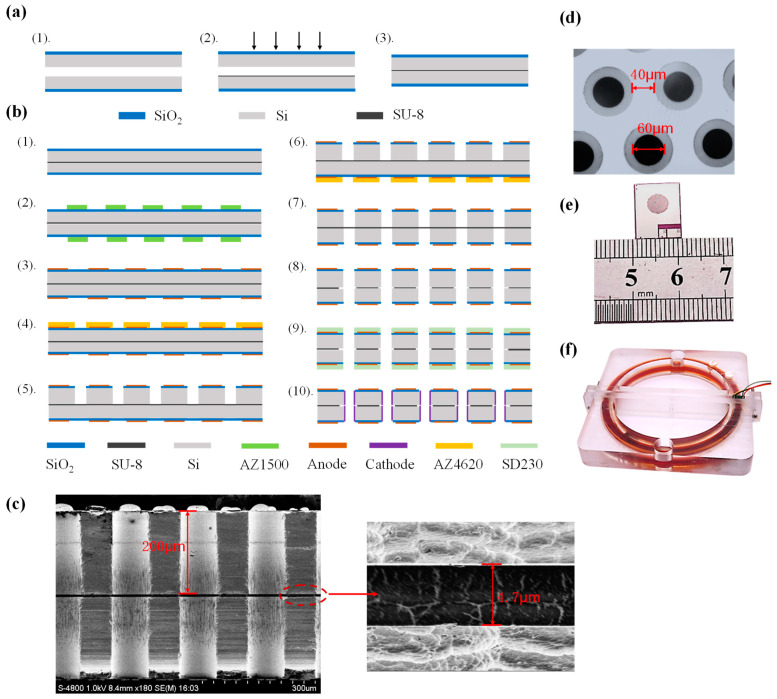
(**a**) Process of wafer-level bonding, including key steps of PECVD, Spin-coating of SU-8, compression and drying. (**b**) MEMS process for fabricating the silicon-based four-electrode structure, including sputtering of anodes, etching of flow channels, etching of SU-8, and sputtering of cathodes. (**c**) The cross-section profile of the flow-through holes by SEM, (**d**) The surface of the fabricated silicon-based four-electrode structure by electron microscope. (**e**) The image of the fabricated silicon-based four-electrode structure. (**f**) The assembled MEMS electrochemical angular accelerometer with the silicon-based four-electrode structure.

The silicon-based four-electrode structure was fabricated by conventional MEMS processes, which were divided mainly into two steps of wafer-level bonding and wafer-level etching. In the step of wafer-level bonding, SU-8 was used as the adhesive layer because of its high insulation and stability to bond two silicon wafers with a thickness of 200 μm and a diameter of 4 inches. The wafer-level bonding based on SU-8 is shown in Figure 3a, which was composed of (i) cleaning and oxidation of silicon wafers with a thickness of 1 μm based on plasma enhanced chemical vapor deposition (PECVD), (ii) Spin-coating of SU-8 photoresist on silicon wafers, and (iii) bonding with alignment enabled by a laminating machine for compression and a hot plate for drying.

The bonded wafers were manufactured into the silicon-based four-electrode structures following the MEMS process shown in Figure 3b, which was described as follows.

Step 1. Washing the bonded silicon wafer by boiling acid and deionized water.
Step 2. Spin-coating AZ1500 on both sides of the bonded silicon wafer, followed by prebaking, lithography, and development to function as a mask for anode sputtering.
Step 3. Sputtering Ti/Pt alloy with a thickness of 300/2500 Å on both sides of the silicon wafer, followed by lift-off to obtain anodes.
Step 4. Spin-coating AZ4620 on the front side of the bonded silicon wafer, followed by prebaking, lithography, and development to function as a mask for front-side etching.
Step 5. RIE etching the front side to remove the 1 μm silicon oxide layer, and DRIE etching the 200 μm silicon substrate to form frontside flow-through holes.
Step 6. Spin-coating AZ4620 on the backside of the bonded silicon wafer, followed by prebaking, lithography, and development to function as a mask for backside etching.
Step 7. RIE etching the backside to remove a 1 μm silicon oxide layer, and DRIE etching the 200 μm silicon substrate to form backside flow-through holes.
Step 8. RIE etching the intermediate SU-8 bonding layer with the etching gas of CF_4_:O_2_ = 1:4 to create fully penetrating flow-through holes.
Step 9. Attaching SD230 photoresist on both sides of the bonded silicon wafer, followed by prebaking, lithography, and development to function as a mask for cathode sputtering.
Step 10. Sputtering Ti/Pt alloy with a thickness of 300/2500 Å on both sides, followed by lift-off to obtain cathodes.

The cross-sectional profiles of the flow-through holes were captured using a scanning electron microscope (SEM), and the locally magnified image confirmed the uniformity of the intermediate layer SU-8, as shown in Figure 3c. It can be clearly seen that through the etching of RIE and DRIE, a through flow hole of 200 μm above and below was fabricated on the silicon-based four-electrode structure of the chip, and the thickness of the intermediate SU-8 layer is 4.7 μm. Figure 3d shows a microscopic view of the flow hole structure of the bonded silicon wafer before dicing. The black part is the flow holes with sidewall cathodes, and the gray part is the silicon dioxide insulation rings, while the white part is the anodes. The electrode structure shown in Figure 3d has a flow hole with a diameter of 60 μm and an anode width of 40 μm. The flow holes are consistent in size and evenly distributed. Figure 3e shows a chip of the silicon-based four-electrode structure after dicing, with a size of 10.4 mm × 13.7 mm. The middle circular part is densely distributed with thousands of flow holes, and cathodes on the sidewalls are led out to the lead hole (on the bottom right) through bulk silicon conduction. The fabricated silicon-based four-electrode structure was then assembled into an enclosure with mechanical compression, followed by the injection and sealing of the electrolyte solution (see Figure 3f). The dark part is an annular flow channel with a diameter of 120 mm. The channel was filled with electrolyte, and the chip was placed in the channel, which was the effective sensing part.

## 5. Results

The characterization system for the fabricated MEMS electrochemical angular accelerometer with the silicon-based integrated four-electrode structure included a standard angular vibration table that can provide sinusoidal vibrations, a DC power supply, a current-voltage conversion circuitry, a data acquisition card, and a LabVIEW-based data processing platform. The standard angular vibration table can provide a frequency signal ranging from 0.01 Hz to 10 Hz, and the corresponding output angular acceleration values at different frequencies can be adjusted by the excitation amplitudes.

**Figure 4:**
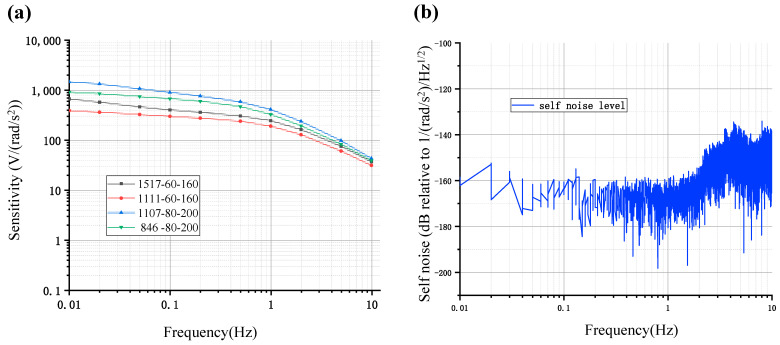
(**a**) Amplitude-frequency characteristic curves and (**b**) self-noise level for the MEMS electrochemical angular accelerometers with the silicon-based four-electrode structure under a group of geometrical parameters (1517-60-160, 1111-60-160, 1107-80-200 and 846-80-200).

The devices were assembled as follows: 1517 (number of flow holes)-60 (diameter of flow holes in μm)-160 (length of cathodes in μm), 1111-60-160, 1107-80-200, and 846-80-200. Note that the length of the insulation layer between anodes and cathodes was 20 μm. The assembled silicon-based integrated four-electrode structures of MEMS electrochemical angular accelerometers were placed on the angular vibration table, and a frequency signal ranging from 0.01 Hz to 10 Hz was provided. During the testing, it usually takes a period of time to stabilize the devices. And the stability of the device is determined by the consistency of multiple repeated tests. The obtained final sensitivity-frequency characteristics curve after stabilization is shown in Figure 4.

The overall trend of the curve indicated a characteristic of a low-pass filter, where the sensitivity gradually decreased as the frequency was increased, which was consistent with the simulation results. Among them, the sensitivities of 1517-60-160 V.S. 1111-60-160 devices at frequencies of 0.01 Hz, 0.1 Hz, and 1 Hz were quantified as 665.7912 V.S. 390.2359 V/(rad/s^2^), 399.5451 V.S. 298.7483 V/(rad/s^2^), and 243.9414 V.S. 190.3566 V/(rad/s^2^), where the differences between these two frequency response curves were stable, respectively corresponding to the black and red lines in Figure 4a. Compared to the 1517-60-160 device and the 1111-60-160 device, they only differed in the number of holes. The 1517-60-160 device had a larger effective area of cathodes and a lower flow resistance due to a larger number of holes, and therefore had higher sensitivity and narrower bandwidth, while the 1111-60-160 device was the opposite. The sensitivities of the 1107-80-200 device vs. the 846-80-200 device at frequencies of 0.01 Hz, 0.1 Hz, and 1 Hz were quantified as 1458.9611 V.S. 905.3151 V/(rad/s^2^), 897.1403 V.S. 671.8375 V/(rad/s^2^), and 407.7017 V.S. 327.0718 V/(rad/s^2^), respectively, corresponding to the blue and green lines in Figure 4a. From the perspective of the number of holes, the 1107-80-200 device had a larger effective area of cathodes and a lower flow resistance than the 846-80-200 device.

Taking into account the bandwidth and sensitivity of the four devices, the 1107-80-200 device had the highest low-frequency sensitivity due to the largest effective cathode area while maintaining a wide frequency band. As the 1107-80-200 device had the best performance among the four devices, subsequent analysis would focus on the 1107-80-200 device.

The 1107-80-200 MEMS electrochemical angular accelerometer with the silicon-based integrated four-electrode structure was then placed on a flat surface and tested for self-noise level at night, where the self-noise power spectral density is shown in Figure 4b. The noise levels at 0.01 Hz, 0.1 Hz, 1 Hz, and 2 Hz were quantified as −161 dB, −164 dB, −162 dB, and −158 dB, respectively. The device exhibited stable low noise levels within the range of 0.01–2 Hz, with slightly increased noise values (lower than −140 dB) in the 2–10 Hz frequency range. Besides the high performance from the perspective of device sensitivity, the MEMS electrochemical angular accelerometer developed in this study also exhibited excellent signal-to-noise ratios.

**Figure 5:**
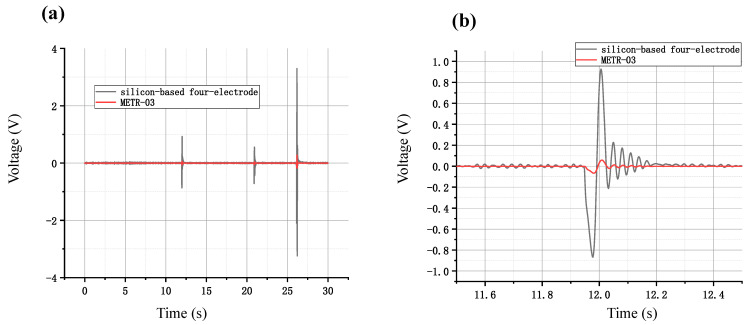
(**a**) Overall and (**b**) local voltage-time curves of the MEMS electrochemical angular accelerometer with the silicon-based four-electrode structure vs. the commercial counterpart of METR-03 in response to artificial vibrations.

The 1107-80-200 MEMS-based electrochemical angular accelerometer was subjected to artificial excitations along with the commercial device METR-03 for seismic monitoring. The test results, as shown in Figure 5a, indicated that both devices clearly captured multiple artificial excitations with consistent response times, where the correlation coefficient between the two devices was 0.9527. The signal amplitude outputted by the device’s silicon-based integrated four-electrode structure was significantly larger than that of the METR-03 device. More specifically, after an artificial excitation at 11.9 s, the maximum amplitude from the device with the silicon-based integrated four-electrode structure was 0.86 V, while the METR-03 device only outputted 0.06 V. This comparison can be observed clearly in the local vibration signal amplification graph, as shown in Figure 5b.

Finally, the performance of the MEMS electrochemical angular accelerometer with the silicon-based four-electrode structure was compared to the previously reported MEMS electrochemical angular accelerometers with a silicon-based three-electrode structure [15], a planar electrode structure [16], and electrochemical angular accelerometers with platinum mesh electrodes [20], as shown in Table 2. Compared to the other three devices, this device had a very high sensitivity of 42 V/(rad/s^2^) at 10 Hz and the lowest noise level of −164 dB. The excellent performance of the silicon-based four-electrode structure is due to the complete and controllable SU-8 intermediate layer, stable differential output, and high on-chip electrode area utilization. The MEMS electrochemical angular accelerometer with the silicon-based four-electrode structure exhibited excellent electrode structures, efficient electrochemical reactions, and a well-developed device packaging process, resulting in noticeable low noise levels and high sensitivities.

## 6. Conclusions

In this study, we present a MEMS electrochemical angular accelerometer with a silicon-based four-electrode structure that is made of thousands of interconnected microchannels for electrolyte flow, anodes uniformly coated on structure surfaces, and cathodes located on the sidewalls of flow holes. The silicon-based four-electrode structure was fabricated by conventional MEMS processes, mainly wafer-level bonding and wafer-level etching. The optimal parameters for the chip were ultimately determined as follows: the number of flow holes is 1107, the diameter of the flow hole is 80 μm, and the length of the cathode is 200 μm. As to device characterization, the MEMS electrochemical angular accelerometer with the silicon-based four-electrode structure exhibited a maximum sensitivity of 1458 V/(rad/s^2^) at 0.01 Hz and a minimum noise level of −164 dB at 1 Hz. The excellent performance of the silicon-based four-electrode structure is due to the complete and controllable SU-8 intermediate layer, stable differential output and high on-chip electrode area utilization. Compared with previously reported electrochemical angular accelerometers with platinum mesh electrodes and silicon-based three-electrode structures, the angular accelerometer developed in this study offered higher sensitivities and lower noise levels, indicating strong potential for applications to monitor and prevent the hazards caused by the rotational components of seismic motions and to comprehensively analyze the causes of earthquakes.

## Figures and Tables

**Table 1:** Normalized flow resistance corresponding to different parameters.

Length of Cross Section	Number of Holes	Diameter of Holes	Normalized Flow Resistance
500 μm	2	220	0.000239
500 μm	3	127	0.001435
500 μm	4	88	0.04669
500 μm	5	52	0.030636
500 μm	6	33	0.157402
500 μm	7	20	1

**Table 2:** Comparison of key parameters of electrochemical angular accelerometers.

Characteristic	Unit	[20]	[15]	[16]	This Device
sensitivity	V/(rad/s^2^)	1 @10 Hz	2.405 @10 Hz	0.033 @10 Hz	42 @10 Hz
noise level	(rad/s^2^)/Hz^1/2^	−105 dB	−154 dB	−118 dB	−164 dB

## Data Availability

Data are contained within the article.
